# The Pancreas as a Target of Metastasis from Renal Cell Carcinoma: Is Surgery Feasible and Safe? A Single-Center Experience in a High-Volume and Certified Pancreatic Surgery Center in Germany

**DOI:** 10.3390/jcm13071921

**Published:** 2024-03-26

**Authors:** Sara Al-Madhi, Sara Acciuffi, Frank Meyer, Maximilian Dölling, Asmus Beythien, Mihailo Andric, Mirhasan Rahimli, Roland S. Croner, Aristotelis Perrakis

**Affiliations:** Department of General, Abdominal, Vascular and Transplant Surgery, Otto-von-Guericke University with University Hospital, Leipziger Str. 44, 39120 Magdeburg, Germany; sara.acciuffi@med.ovgu.de (S.A.); f.meyer@med.ovgu.de (F.M.); asmus.beythien@st.ovgu.de (A.B.); mihailo.andric@med.ovgu.de (M.A.); mirhasan.rahimli@med.ovgu.de (M.R.); roland.croner@med.ovgu.de (R.S.C.); aristotelis.perrakis@med.ovgu.de (A.P.)

**Keywords:** spectrum of pancreas-associated tumor (-like) lesions, secondary malignant tumors of the pancreas, pancreas malignancy, pylorus-preserving pancreatoduodenectomy (PPPD)

## Abstract

**Background**: Secondary malignant tumors of the pancreas are rare, representing 2–5% of all pancreatic malignancies. Nevertheless, the pancreas is one of the target organs in cases of metastatic clear cell renal cell carcinoma (CCRCC). Additionally, recurrent metastasis may occur. Surgical resection remains the best and prognostically most favorable therapeutic option in cases of solitary pancreatic metastasis. Aim: To review retrospectively the clinical tumor registry of the University Hospital of Magdeburg, Germany, for this rare entity, performing a clinical systematic single-center observational study (design). **Methods**: A retrospective cohort analysis of consecutive patients who had undergone pancreatic resection for metastatic CCRC was performed in a single high-volume certified center for pancreatic surgery in Germany from 2010 to 2022. **Results**: All patients (*n* = 17) included in this study had a metachronous metastasis from a CCRCC. Surgery was performed at a median time interval of 12 (range, 9–16) years after primary resection for CCRCC. All 17 patients were asymptomatic at the time of diagnosis. Three of those patients (17.6%) presented with recurrent metastasis in a different part of the pancreas during follow-up. In a total of 17 patients, including those with recurrent disease, a surgical resection was performed; Pancreatoduodenectomy was performed in 6 patients (35%); left pancreatectomy with splenectomy was performed in 7 patients (41%). The rest of the patients underwent either a spleen-preserving pancreatic tail resection, local resection of the tumor lesion or a total pancreatectomy. The postoperative mortality rate was 6%. Concerning histopathological findings, seven patients (41%) had multifocal metastasis. An R0 resection could be achieved in all cases. The overall survival at one, three and five years was 85%, 85% and 72%, respectively, during a median follow-up of 43 months. **Conclusions**: CCRC pancreatic metastases can occur many years after the initial treatment of the primary tumor. Surgery for such a malignancy seems feasible and safe; it offers very good short- and long-term outcomes, as indicated. A repeated pancreatic resection can also be safely performed.

## 1. Introduction

In Germany, the incidence of renal cell carcinoma (RCC) is around 2–3% of all malignant tumors, according to the statistical report from the Robert Koch Institute for the year 2018 [1]. RCC is the most common type of renal cancer, accounting for 85% of all kidney tumors. Clear cell renal cell carcinoma (CCRCC) represents 75–80% of RCC cases [1,2]. Approximately 16% of CCRCC patients are diagnosed with synchronous metastases (mCCRCC) at the time of diagnosis-finding. Around 30–50% of patients with CCRCC develop metachronous metastatic disease during follow-up after initial treatment with curative intention [3,4]. Pancreatic metastasis is rare and occurs mostly in a metachronous fashion after the initial diagnosis of CCRCC. Disease recurrence can occur decades after the primary diagnosis. Metastasis to the pancreas, when it occurs, is considered a late-relapsing disease, which indicates longer survival [5]. This can sometimes be misdiagnosed as primary pancreatic cancer. Therefore, it is of utmost importance to perform a thorough diagnostic workup for these patients to achieve an accurate diagnosis. Unlike primary pancreatic cancer, it has been shown that patients with CCRCC pancreatic metastasis undergoing resection have a favorable prognosis, with a five-year survival rate of about 75% [3,6]. Nevertheless, due to the rarity of the disease, a few reports or case series were presented in the literature. The surgical option, particularly in cases of single-site metastasis among surgically fit patients, is considered to be a valuable and only curative choice in the treatment of mCCRCC, irrespective of the site of the metastasis. Therefore, surgical resection of the metastasis is expected to enhance disease-free survival [7].

Recent advances, evolution and new approaches to the management of metastatic disease have evolved over the years. These include advancements in surgical techniques and perioperative management setups, the emergence of state-of-the-art chemotherapy, immunotherapy like tyrosine kinase inhibitors and improvements in interventional radiology. 

In recent years, combination immunotherapy regimes like axitinib and pembrolizumab, approved in 2019, have shown significant advancement. This combination has demonstrated superiority over sunitinib in terms of overall response rate and progression-free survival (PFS). Despite the advances in targeted therapy, achieving a complete response and cure for mCCRCC through systemic treatment alone remains rare [7,8,9,10].

A deeper understanding of the disease and the appropriate management plan, along with the recognition of the effectiveness and limitations of each treatment method in an interdisciplinary setting, are of great importance.

In the domain of pancreatic surgery, a notable evolution in surgical techniques over the years has been observed. The centralization of pancreatic surgery has significantly improved the postoperative mortality rate, which ranges between 2% and 8% [11]. The incidence of postoperative overall morbidity has also decreased but still remains high, between 30% and 60% [9]. 

Therefore, after extensive review of the Clinical Tumor Registry of the University Hospital of Magdeburg (Germany), we retrospectively analyzed overall 17 patients who had undergone surgical resection for CCRCC pancreatic metastasis in a university hospital setting (i.e., highly specialized care—e.g., characterized by a high case volume of a certified “Pancreas Cancer Center”) in Germany between 2010 and 2022.

## 2. Materials and Methods

### 2.1. Study Design 

A prospectively acquired, single-center database from the University Hospital of Magdeburg, Germany, was retrospectively analyzed.

The following is a retrospective analysis of consecutive patients who underwent pancreatic resection for mCCRCC at the Department of General, Abdominal, Vascular and Transplant Surgery, University Hospital of Magdeburg (Germany), from January 2010 to May 2022. After extensive laboratory, endoscopic and radiological workup, including computed tomography (CT) and/or magnetic resonance imaging (MRI) in the follow-up after resection for CCRCC, all patients underwent a fine needle biopsy before indication for surgery. The diagnosis of oligometastatic renal carcinoma was confirmed in all patients (as one of the crucial inclusion criteria) included in the study group. All of these patients previously underwent curative treatment for localized renal cell carcinoma. Patients who had undergone previous R0 metastatic resections in other organs such as the thyroid, lungs and liver were also included. Excluded from this study were patients with primary pancreatic cancer or pancreatic invasion resulting from other malignancies, as well as patients with multiple metastatic sites who were undergoing systemic therapy. Moreover, patients who were not fit for surgery and who received local interventional radiological therapy were also excluded due to their limited number.

### 2.2. Surgical Procedure 

All surgical procedures (both open and minimally invasive) for this entity were performed by a surgical team with a high level of experience in pancreatic surgery (over 25 pancreatic resections per year for each surgeon). None of those patients had an invasion of the adjacent vessels. All types of pancreatic resections, such as typical pancreatic resection including pylorus-preserving Pancreatoduodenectomy (PPPD), left pancreatectomy (LP) with splenectomy, pancreatic body resection (PBR), total pancreatectomy (TP) and atypical resection, such as enucleation, were performed for mCCRCC patients included in the current analysis. A standard lymphadenectomy was performed, and an intraoperative frozen section was obtained in all cases to ensure a resection-free margin.

### 2.3. Collection of Clinical Data

We conducted a comprehensive analysis including all demographic variables (e.g., age, symptoms, body mass index [BMI]). The preoperative evaluation of patients’ eligibility for surgery involved assessments according to the Eastern Cooperative Oncology Group (ECOG) and according to the American Society of Anesthesiologists (ASA score) [12,13]. In addition, clinical prognostic factors or risk models for metastatic renal cell carcinoma according to the International Metastatic Renal Cell Carcinoma Database Consortium (IMDC) were used [14]. These factors include laboratory values such as calcium, hemoglobin and neutrophil levels, as well as the Karnofsky performance status to categorize patients into favorable (0 points), intermediate risk groups (1–2 points) and poor prognostic groups ≥ 3 (Table 1).

Furthermore, the analysis considered the following factors:

− Comorbidities;
− The stage of the primary renal tumor;
− The duration between the primary renal surgery and pancreatic metastasis;
− The presence of other metastases;
− Whether the pancreatic metastasis was primary or recurrent.

Perioperative outcomes, including the length of hospital and intensive care unit (ICU) stays, surgical techniques employed, intraoperative blood loss and operating time, were also included in the analysis. Postoperative complications were classified according to the Clavien–Dindo classification [15]. Regarding postoperative morbidity, we assessed the postoperative surgery-related complications, such as postoperative pancreatic fistula, hemorrhage, lymphatic fistula and delayed gastric emptying. This assessment was based on the classification criteria defined by the International Study Group of Pancreatic Surgery (ISGPS) [16]. The analysis involved macroscopic pathology as well as histological examination data, such as the size of metastasis, the presence of multifocal pancreatic metastases and the evaluation of surgical margins. For follow-up information, we conducted a retrospective analysis of medical reports and established direct contact with the patients or their primary health care providers over a median period of 43 months.

### 2.4. Statistical Analysis and Ethical Approval

Electronic data collection was conducted using Redcap^®^ (Research Electronic Data Capture, version number 11.1.1 © 2024 Vanderbilt University) under a university license in cooperation with the local Department of Information Technology (IT). 

Statistical analysis was carried out using RedCap^®^ (Vanderbilt University; Nashville, TN, USA) and IBM^®^ SPSS Statistics software (BM SPSS Statistics; version number 29.01 Chicago, IL, USA). The calculation of the overall and recurrence-free survival rates was carried out using the Kaplan–Meier method. All procedures performed were in accordance with the ethical standards of the institutional and/or national research committee and with the 1964 Helsinki Declaration for Biomedical Research of the “World Medical Association” and its later amendments. This paper aligns with the recommendations of the PROCESS guidelines [17].

## 3. Results

**Figure 1:**
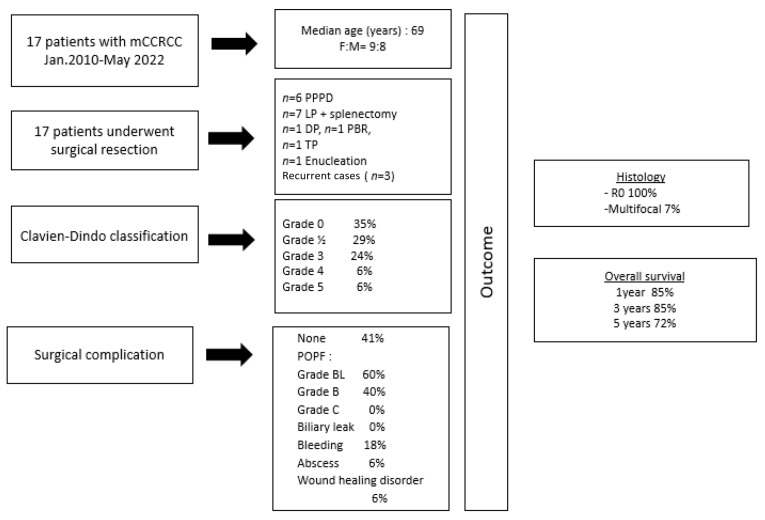
Overview of the study characteristics (*n* = 17 patients).

Surgery was performed on a total of 17 patients with pancreatic metastasis of CCRCC. In terms of demographic data (Table 1, Figure 1), the sex ratio was F:M = 9:8, with a median age of 69 (range, 57–82) years. The median BMI was 28.7 (range, 18.3–41.8) kg/m^2^. All patients who qualified for surgery had an acceptable ECOG status (ECOG, 0–1). However, 53% of them were categorized as having a higher ASA score (>2). Within our case series, the majority of patients (78%) were asymptomatic, while 22% presented with non-specific symptoms, such as body weight loss exceeding 10 kg in the 6 months preceding surgery. Additionally, over 50% of the patients had type 2 diabetes before surgery (Table 1). According to the IMDC score, none of the patients had a Karnofsky performance status of less than 80% (*n* = 0/17) at the time of assessment. Moreover, none of the patients received systemic treatment following diagnosis (*n* = 0/17). Among the cohort, two patients presented with hemoglobin levels below the lower limit of normal (LNL) (*n* = 2/17), while neutrophil levels were within the normal range for all patients (*n* = 17/17). Additionally, one patient demonstrated platelet counts at the upper limit of normal (UNL) (*n* = 1/17), and another patient had calcium levels at the upper limit of normal (*n* = 1/17).

A total of 14 patients had 0 prognostic factors (*n* = 14/17), which indicate a favorable risk; 2 patients had 1 prognostic factor (*n* = 1/17) and 1 patient had 2 prognostic factors (*n* = 2/17), which indicate intermediate risk. None of our cohorts exhibited three or more of these factors.

Clinical data concerning the primary tumor are demonstrated in Table 2. Most of the patients had their primary tumor on the left side (59%). All patients underwent initially curative-intended surgery for the primary tumor, including the following:

− Nephrectomy in 72% of cases;
− Multivisceral resection (12%);
− Partial kidney resection (6%).

**Table 2:** Characteristics of the primary tumor lesion.

Characteristics	Number	%
Histology		
CCRCC	17	100
Other	0	0
Multifocal	7	41.2
Lymph node involvement	0	100
Side of primary		
Left	10	59
Right	7	41
Resection of primary		
Partial resection	1	6
Nephrectomy	14	72
Multivisceral resection	2	12
Previous pancreatic metastases/resection	3	18
Timing		
Metachronous	17	100
interval from onset of CCRCC to PM (median, months)	154	

CCRCC, clear cell renal carcinoma; PM, pancreatic metastasis.

None of the patients received chemotherapy or immunotherapy prior to surgery. All patients were diagnosed with metachronous metastasis in a median interval of 154 (range, 12–239) months after the initial diagnosis. Within the investigated cohort, three patients presented with recurrent pancreatic metastasis:

− Two had previously undergone PPPD;
− One had undergone an LP.

Pancreatic metastasis was the initial localization of metastatic disease in seven cases (41%). The rest of the patients had a metastatic disease involving other organs, such as the following:

− Liver (*n* = 2);
− Suprarenal gland (*n* = 2);
− Spleen (*n* = 1);
− Breast (*n* = 1);
− Lung (*n* = 3);
− Thyroid gland (*n* = 3).

All patients with additional metastatic localizations had previously undergone an R0 resection for metastasis and were under regular follow-up care.

Concerning the surgical approach to the pancreatic metastasis, a local excision/enucleation of the tumor lesion was performed in one case, while the remaining 16 patients underwent a formal pancreatic resection (Table 3 and Figure 1):

− LP (*n* = 7);
− PPPD (*n* = 6);
− Pancreatic tail resection (*n* = 1);
− PBR (*n* = 1);
− TP (*n* = 1).

**Table 3:** Operative data (including the three recurrent cases).

Characteristics	Number	%
Surgical intervention		
-Enucleation	1	6
-PPPD	6	35
-Pancreatic tail resection	1	6
-Left pancreatectomy with splenectomy	7	41
-Pancreatic body resection	1	6
-Total pancreatectomy	1	6
Open vs. MIS		
-Open	16	94
-MIC	1	6
Operating time (median, min)	211	
Blood loss (median, mL)	350	
Infusion of crystalloid (median, mL)	3500	
Blood infusion		
-Yes	4	23
-No	13	77

PPPD, Pylorus-preserving pancreatoduodenectomy; MIS, minimally invasive surgery.

Three patients experienced recurrent pancreatic metastasis, leading to repeated pancreatic resections. The patient who initially had undergone PPPD, experienced a recurrence in the body of the pancreas, leading to a subsequent resection of the body of the pancreas. Another patient, who had previously undergone PPPD, underwent a TP due to multiple recurrent pancreatic metastases. The third patient, who initially had undergone a pancreatic tail resection, was indicated for a subsequent procedure in terms of LP. Among this group of patients, one patient experienced wound infection following repeated surgery. However, no major surgical complications were observed, and there were no non-surgical complications noted.

The median duration of the operative procedure was 211 (range, 90–340) min, with a median blood loss of 350 (range, 100–600) mm. Blood transfusions were required in four patients. The median length of in-hospital stay was 12 (range, 9–16) days, while the median length of ICU stay was 2 (range, 1–8) days (Table 3 and Table 4).

As far as postoperative morbidity is concerned, five patients had the diagnosis of a postoperative pancreatic fistula. Only two of them were classified as grade B POPF. Additionally, we observed three incidents of postoperative bleeding and one intra-abdominal postoperative abscess. No perioperative surgery-related mortality was recorded. One patient died due to pneumonia five weeks after surgery. In total, six patients (35%) experienced postoperative morbidity with a Clavien–Dindo score greater than 3 and a need for intervention (either re-surgery [*n* = 1] or radiological intervention [*n* = 5]). In four cases (24%), a new onset of type 3 diabetes was observed in the postoperative setting (Table 4 and Figure 1).

In terms of postoperative histopathology, in all cases, an R0 resection and the occurrence of metastatic CCRCC could be confirmed. Among the patients, seven patients (41%) presented with multifocal lesions. No lymph node metastasis was detected (Table 5 and Figure 1).

**Figure 2:**
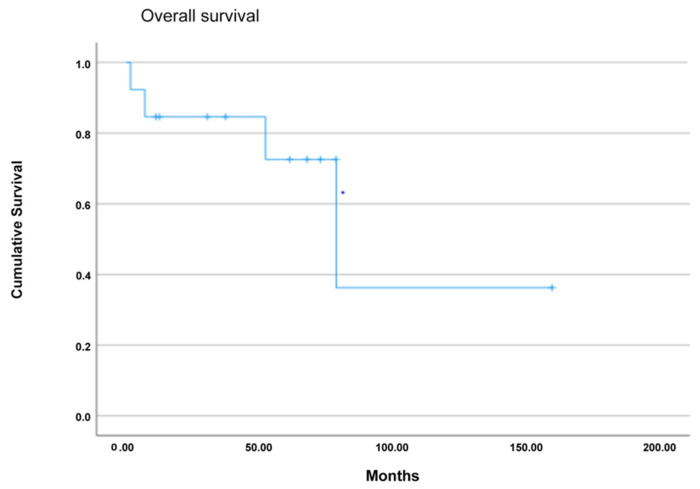
Kaplan–Meier survival curve of 5-year overall survival (OS) following pancreatic resection.

**Figure 3:**
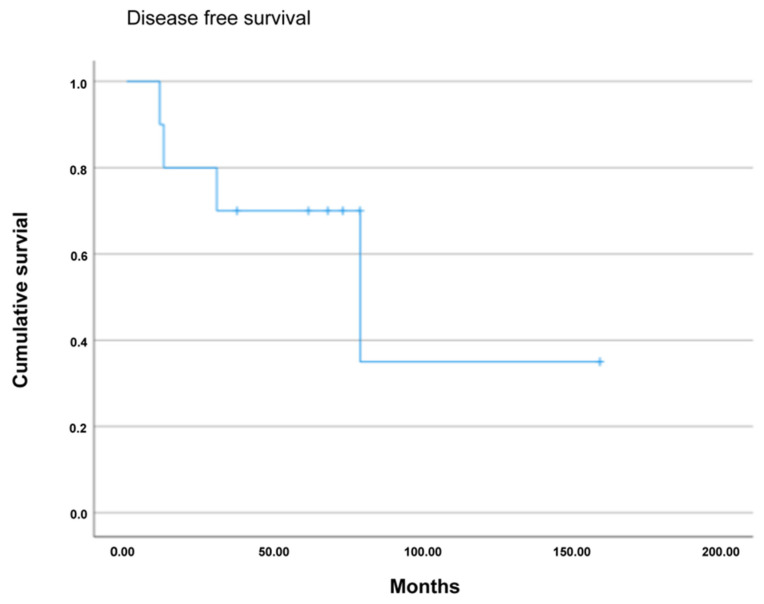
Kaplan–Meier survival curve of 5-year disease-free survival (DFS) following pancreatic resection.

The median follow-up time was 43 (range, 12–75) months. The overall survival at one, three and five years was 85%, 85% and 72%, respectively. The disease-free survival at one, three and five years was 80%, 70% and 70%, respectively. Six patients experienced a relapse of the disease during follow-up after a median time period of 35 (range, 20–50) months (Figure 2 and Figure 3).

## 4. Discussion

Patients diagnosed with CCRCC can develop metastasis either in a synchronous or metachronous setting in approximately 25% of the cases. The most common metastatic sites are the lungs, bones, liver and brain. However, the pancreas might also be a possible site of metastasis. The reported incidence varies from 1.6% to 11% in autopsy studies and 2% to 5% in clinical studies [18,19]. This metastasis typically manifests in a metachronous fashion and, in numerous cases, often occurs several years after primary tumor treatment [20,21]. Due to the rarity of this kind of metastatic pattern, clinical studies concerning the treatment of CCRCC metastasis in the pancreas are currently lacking.

As observed in our study, the majority of patients presented within twelve years after the initial diagnosis, highlighting the necessity of long-term surveillance.

Early detection of metastasis is associated with a favorable prognosis, leading to a significantly longer overall survival, unlike the late-diagnosed metastatic pattern, which is associated with poor five-year survival rates [22,23].

In general, pancreatic metastasis (PM) is rare, comprising 2–5% of all pancreatic malignancies [6,22]. The pathway of RCC metastasis as well as the tumor biology of the metastasis remain poorly understood [24,25]. The clinical presentation is often non-specific, and the detection of metastasis typically occurs during follow-up after resection of the primary tumor. In the case series presented here, a majority of patients were asymptomatic, with tumor detection taking place during routine follow-up. This aligns with findings from a recent systemic review by Huang et al., who emphasized the importance of routine follow-up with these patients [26].

The management of mCCRCC has been developed with the introduction of different systemic agents. However, these therapies alone rarely achieve a complete response [7,27]. Many reports have shown that a resection of the primary tumor with surgical resection of the metastasis in well-selected patients has been associated with a better survival outcome [7,9]. Unfortunately, due to the small number of patients and the uniqueness of this type of metastasis, there is no randomized control trial available to determine the benefits. Shin et al. reported on pancreatic metastasis resection outcomes, demonstrating an improvement in overall survival with metachronous pancreatic metastases [28].

Surgical resection of oligometastatic CCRCC has been a standard treatment, according to multiple studies showing a survival benefit for surgical resection of all metastatic lesions, resulting in significant survival prolongation in eligible patients [4,6,29,30]. However, the management of mCCRCC remains complex and challenging. As far as surgery for mCCRCC is concerned, several studies reported a postoperative mortality rate ranging from 1.6% to 4.1% and a morbidity rate of 18% [31]. However, in the era of immunotherapy, targeted therapy and checkpoint inhibitors, the role of surgery has continuously been questioned and challenged. Many retrospective studies have repeatedly demonstrated the benefit of complete metastasectomy on survival; even the incomplete removal of metastatic lesions has been shown to improve survival as well [9,28].

Over the past decade, there has been tremendous progress in various aspects of pancreatic surgery. Developments in surgical techniques have led to improved postoperative outcomes, which can be noticed by a significant reduction in postoperative morbidity. Furthermore, enhanced perioperative management and the implementation of ERAS (enhanced recovery after surgery) concepts in pancreatic surgery have contributed to better surgical outcomes, resulting in a decrease in 30-day mortality rates [27].

Advancements in the efficacy and safety of pancreatic surgery, particularly in high-volume centers, with the introduction of minimally invasive surgical approaches have significantly improved surgical outcomes [25]. Postoperative complications, particularly pancreatic fistulas, represent a significant concern after pancreatic resections. The incidence of postoperative pancreatic fistulas reported in the literature varies widely, ranging from 8% to 30%, depending on the definition and grading criteria used. In a study conducted by Lee et al. involving 98 patients who underwent pancreatic resection for pancreatic metastasis from different primary cancers, the reported postoperative morbidity and mortality rates were 56% and 3.1%, respectively [32]. The postoperative morbidity and mortality rates for pancreatic adenocarcinoma following pancreatic resection were found to be nearly equivalent, indicating acceptable outcomes in the surgical treatment of mRCC [33,34]. In the case series presented here, the postoperative morbidity rate was acceptable and comparable to other series [33,34]. In total, only six patients experienced postoperative morbidity with a need for intervention, mostly in the fashion of interventional radiology, such as CT-guided placement of a (pigtail or rinsing) drainage (*n* = 5). Therefore, advanced pancreatic surgery should be conducted at “high-volume” centers led by experienced HPB (hepatopancreatobiliary) surgeons. It should be emphasized that the availability of expert interventional radiology plays an important role in effectively managing morbidity and its significant implications.

Regarding the surgical techniques and a recommendation on whether to perform organ-preserving resection, such as enucleation or extended resection for pancreatic metastasis, clear evidence is currently lacking. Bassi et al. reported a rate of pancreatic recurrences after performing organ-preserving procedures of up to 29% [25]. Nevertheless, metastasis from RCC is mostly multifocal and can occur in 20–40% of the cases [6,35]. Therefore, it is still not clear if such recurrences after enucleation are due to an inadequate surgical approach or to undetected multifocal disease. Zerbi et al. suggested that an adequate local excision is not associated with a higher recurrence compared to standard resection, provided that an accurate preoperative diagnostic workup is the key to adequate preoperative planning [35]. In our case series, seven patients (41.2%) presented with multifocal lesions. This observation may support the preference for extended oncological resection over local excision.

Another interesting point is the involvement of the local peripancreatic lymph node regions and the need for radical lymph node dissection. We prefer the radical surgical approach with lymphadenectomy—as performed for pancreatic adenocarcinoma—since the presence of lymph node metastasis is associated with a poor prognosis [36]. In the present study, no lymph node involvement was noticed. This is also supported by other clinical studies [5,37].

The most common surgical approach in our case series was open surgery. Only one patient underwent a minimally invasive LP. Various surgical approaches have been described, including open and minimally invasive procedures. The choice of surgical technique depends on factors such as tumor location, size and the involvement of adjacent structures. Minimally invasive techniques, such as laparoscopic or robotics-assisted, have also been reported with favorable outcomes, including reduced blood loss, shorter hospital stays and comparable oncological outcomes [38,39].

However, our study included patients with mCCRCC over a time period of 12 years, where a minimally invasive surgical approach was not yet established for pancreatic surgery.

Considering survival rates and tumor biology, RCC has a prolonged disease-free interval due to its distinctive characteristics of slow progression [34,40]. The five-year survival rate in resected patients can reach up to 88% (22–88%) [5,41].

In our case series, the overall survival at one, three and five years after surgery was 85%, 85% and 72%, respectively. The disease-free survival at one, three and five years was 80%, 70% and 70%, respectively, and, therefore, comparable to other studies [41].

The recent introduction of systemic therapy regimens such as biologicals and systemic treatment with tyrosine kinase inhibitors, combined immunotherapies and checkpoint inhibitors has been intensively discussed as an alternative treatment option for mCCRCC [5,7]. In a multicenter study comparing surgically treated patients with those treated with tyrosine kinase inhibitors, similar short-term results were found. However, as far as long-term outcomes are concerned, patients who had undergone surgery for mCCRCC had significantly better survival rates in terms of overall and disease-free survival [5]. Nevertheless, the majority of the available randomized controlled trials in the literature have primarily focused on multiple metastatic sites or those who have progressed during or after treatment [42]. For those patients who initially underwent curative resection of the primary tumor and then later developed single-site metastases in their follow-up, the option of surgical resection of the metastasis should always be discussed, especially in surgically fit patients, as survival benefits with metastatectomy have been well-addressed in the literature [14,21,41]. This group of patients has been demonstrated in our case series.

Despite the availability of various systemic therapy options for the management of mCCRCC, the surgical approach represents a significant advantage for patients with toxicities associated with systemic therapies, especially for those with resectable oligometastases. This proves that the surgical option does not compromise overall survival and preserves treatment-free survival [7,43]. Recently, the International Metastatic Renal Cell Carcinoma Database Consortium (IMDC) score has become an important tool used to predict individual patient prognosis. The prognostic index categorizes patients with mCCRCC into three subgroups: good prognosis, characterized by longer survival; intermediate prognosis, with a median overall survival (OS) of approximately 23 months; and poor prognosis, with a median survival of around 8 months [44]. In our patient cohort, 14 patients had a favorable or good score, which indicates long survival.

The *strength* of this study is that surgical clinical studies concerning patients with RCC-metastasis in the pancreas with a long follow-up are missing in the current literature.

On the other hand, this study has several *limitations*. It is a retrospective analysis of an indeed prospectively acquired database, with a relatively small number of patients included. However, this number seems to be respectable, considering the rarity of the reported disease. Furthermore, similarly to prior published data, the absence of a control group is another aspect of a potential limitation. So far, there is no standard approach to the treatment of pancreatic metastasis of CCRCC due to the absence of a prospective clinical trial, which can be explained by the rarity of this type of metastasis.

## 5. Conclusions

In conclusion, these data represent a single-center experience in Germany. Pancreatic resection for metastatic RCC in carefully selected patients, even in recurrent metastasis, and redo surgery remain the best options to provide, ensure or improve long-term outcomes. However, the decision to perform pancreatic resection should be individualized, taking into account patient characteristics, tumor factors and the expertise of the surgical team. A multidisciplinary approach is crucial to the management of metastatic RCC.

## Figures and Tables

**Table 1:** Demographic data of the study cohort, including the risk factors according to IMDC.

Characteristics	Number	%
Median Age [years]	69	
Sex (F:M)	9:8	
Median BMI [kg/m^2^]	28.7	
ASA score		
1–2	8	47
>2	9	53
ECOG Status		
0–1	17	100
>1	0	0
Symptoms	2	22
Preoperative diabetes	7	50
Smoker	3	18
Risk factors (IMDC)		
Karnofsky Performance status < 80%	0	
Time from diagnosis to treatment < 1 year	0	
Hemoglobin level < LNL	2	
Neutrophol level > UNL	0	
Palatelet counts > UNL	1	
Calicum level > UNL	1	
Number of prognostic factors		
0 prognostic factor	14	Favourable risk
1 prognostic factor	2	Intermediate risk
2 prognostic factors	1	Intermediate risk

Abbreviations: ASA, American Society of Anesthesiologists; ECOG, Eastern Cooperative Oncology Group; IMDC, International Metastatic Renal Cell Carcinoma Database Consortium; UNL, upper normal level; LNL, lower normal level.

**Table 4:** Postoperative data.

Characteristics	Number	%
LOS (median, days)	12	
Length of ICU stay (median, days)	2	
Complication (according to the Clavien–Dindo classification)		
Grade 0	6	35
Grade 1/2	5	29
Grade 3	4	24
Grade 4	1	6
Grade 5	1	6
Surgical complication		
None	7	41
POPF	5	29
Grade BL	3	60
Grade B	2	40
Grade C	0	0
Biliary leak	0	0
Bleeding	3	18
Abscess	1	6
Wound healing disorder	1	6
General complication		
None	12	70
Urinary infections	3	18
Pneumonia	1	6
Pulmonary artery embolism	1	6
Postoperative diabetes, type III		
-Yes	4	24
-No	13	76

LOS, length of stay; ICU, Intensive Care Unit; POPF, postoperative pancreatic fistula.

**Table 5:** Postoperative histopathological data of the pancreatic tumor lesions.

Characteristics	Number	%
CCRCC	17	100
Resection margin		
R0	17	100
R1	0	
R2	0	
Rx	0	
Multifocal, unifocal or diffuse		
Multifocal	7	41.2
Unifocal	10	58.8
Diffuse	0	
Lymph node status		
Positive lymph node	0	
Negative	15	88.2
Unknown	2	11.8

CCRCC, clear cell renal carcinoma.

## Data Availability

All relevant data are provided in the manuscript.

## Abbreviations

ASA	American Society of Anesthesiologists
BMI	body mass index
CCRCC	clear cell renal cell carcinoma
ECOG	Eastern Cooperative Oncology Group
HPB	hepatopancreatobiliary
ISGPS	International Study Group of Pancreatic Surgery
LP	left pancreatectomy
mCCRCC	metastatic clear cell renal cell carcinoma
MIS	Minimally invasive surgery
PBR	pancreatic body resection
PPPD	Pancreatoduodenectomy
RCC	renal cell carcinoma
RKI	Robert Koch-Institute
TP	total pancreatectomy
PM	pancreatic metastasis
LOS	length of stay
POPF	postoperative pancreatic fistula
IMDC	International Metastatic Renal Cell Carcinoma Database Consortium
UNL	upper normal level
LNL	lower normal level
